# Interactive effects of silicon formulations, concentrations, and foliar application timing on rice physiology and yield

**DOI:** 10.3389/fpls.2025.1723079

**Published:** 2026-01-21

**Authors:** Elena Velasco, Xavier Aranda, Frank Houben, Juantxo Ribes, Jose L. Araus, Pedro García-Caparros

**Affiliations:** 1 Section of Plant Physiology, Faculty of Biology, University of Barcelona, Barcelona, Spain; 2 Experimental Fields Service, Faculty of Biology, University of Barcelona, Barcelona, Spain; 3 Aptus-Holland, Nuth, Netherlands; 4 AGROTECNIO (Center for Research in Agrotechnology), University of Lleida, Lleida, Spain; 5 Institute of Biogeosciences, IBG2: Plant Sciences, Forschungszentrum Jülich GmbH, Jülich, Germany

**Keywords:** biostimulant, carbon isotope composition, grain elemental composition, nitrogen isotope composition, *Oryza sativa*, vegetation indices

## Abstract

The continuous increase in the cost of water and fertilizers associated with increasing global demand for food driven by population growth and the growing concern on the current environmental impact of agriculture led us to the urgent search for more sustainable agronomic practices. Among these, the use of biostimulants has emerged as a promising strategy to enhance crop productivity and resource-use efficiency while reducing reliance on conventional inputs. Nevertheless, identifying the most suitable type of biostimulant, along with the optimal method, dosage, and timing of application, remains particularly critical for staple crops such as rice, being an area that requires further in-depth research. In the present experiment, two silicon-based biostimulant formulations were tested under controlled conditions at two different concentrations and applied at different key phenological stages in rice through foliar spraying. Agronomical components (plant height, tiller number, aerial dry weight, grain yield, and harvest index), whole plant physiological parameters (vegetation indices such as NGRDI, TGI, GA and GGA readings), leaf traits (photosynthetic and transpirative gas exchange, total nitrogen and carbon concentration and the stable isotopic composition, pigment content), and the grains characteristics (mineral composition (macronutrients and heavy metal concentrations) were evaluated. Among the tested products, the Simosa formulation was the most effective, significantly enhancing tiller number, aerial dry weight, grain yield, chlorophyll concentration and nitrogen balance index. Nevertheless, no consistent dose-dependent effects were observed. In contrast, Siliforce-4 did not demonstrate clear effects on either biomass accumulation or physiological traits. Regarding rice grain consumption, only copper concentrations exceeded the threshold established by EFSA, 2009. Overall, these results underscore the need for further studies to determine the most effective silicon foliar fertilizer formulations, as well as optimal dose and timing of application for boosting rice productivity.

## Introduction

Biostimulants represent a broad category of diverse compounds, encompassing both substances and microorganisms, that exert beneficial effects on plant growth, yield and chemical composition, while simultaneously enhancing the resilience to biotic and abiotic stresses (Shahrajabian et al., 2021; Rouphael and Colla, 2020). Within the current regulatory framework, biostimulants are systematically sorted into two principal groups: microbial and non-microbial. The microbial classification encompasses organisms such as beneficial fungi and bacteria, while the non-microbial category encompasses a diverse spectrum of substances, including plant and seaweed extracts, biopolymers, protein hydrolysates, amino acids, humic acids, and minerals such as silicon (E.U., 2019).

Although silicon (Si) is regarded as a non-essential element, it plays a crucial role in several physiological processes that contribute to enhanced plant growth and development (Souri et al., 2021; Khan et al., 2023). Silicon-based products are available in solid and liquid formulations. Solid Si products are mainly derived from geological sources such as rocks and sediments, as well as from recycled materials, whereas liquid formulations typically contain monosilicic or polysilicic acid solutions (Zellner and Datnoff, 2020; Mathur and Roy, 2020). These products can be supplied either through soil incorporation or foliar applications. Foliar application offers significant advantages, particularly in circumventing potential constraints linked to immobilization of Si in soil and is therefore preferred in scenarios that require repeated sprays targeted to specific plant organs. In the context of foliar uptake, silicon can be absorbed directly through the cuticle or via specialized openings on the leaf surface, including clefts adjacent to trichomes, stomata, pores and hydathodes. This mode of uptake highlights the versatility and effectiveness of foliar application as a strategy for targeted Si delivery within agricultural systems (Parimala and Singh, 2022; Garcia-Caparros et al., 2025). The application of silicon plays a vital role in preventing or reducing lodging in cereal crops, thereby enhancing photosynthetic efficiency, which consequently leads to greater biomass accumulation, higher yield, and improved nutrient uptake (Chack et al., 2023).

The integration of remote sensing approaches with targeted laboratory analysis, such as stable isotope profiling, generates a valuable framework for improving the precision and predictive capacity of crop phenotyping (Gracia-Romero et al., 2019; Rezzouk et al., 2020a). Stable isotope ratios of carbon and nitrogen in crop tissues function as a time-integrated markers of physiological responses and interactions with both biotic and abiotic environmental stresses (Dong et al., 2022; Rezzouk et al., 2022). Carbon isotope composition(δ^13^C) when analyzed in dry matter offers insight into the long-term balance between intercellular and atmospheric CO_2_ concentrations (Ci/Ca) during photosynthesis (Farquhar and Richards, 1984; Farquhar et al., 1989; Araus et al., 2003). Total carbon content in plant biomass further represents the extent of atmospheric CO_2_ assimilation through chloroplast-mediated carbon fixation. Similarly, nitrogen isotope composition (δ^15^N), when considered alongside total nitrogen content, provides a dual metric that captures the influence of growth conditions on plant nitrogen uptake and metabolism (Rezzouk et al., 2020b).

Rice is considered one of the most important global staple crops being cultivated across many regions and under different climatic conditions throughout the year. Nevertheless, the consequences of global climate change are rather evident within the rice sector (Samal et al., 2022; Lu, 2024). Extreme weather events, coupled with the heightened incidence of both abiotic and biotic stresses, are directly compromising rice yield and grain quality (Mohapatra and Sahu, 2021; Zhao et al., 2022). Consequently, the development of sustainable strategies to ensure rice production and maintain adequate yield has emerged as one of the most pressing challenges in the recent agriculture. Among the different approaches explored, the application of biostimulants, particularly silicon, has been widely reported in experimental studies as an effective strategy to palliate these constrained conditions (Deus et al., 2020; Jiang et al., 2022; Chan-In et al., 2024; Jin et al., 2024; Kumar et al., 2025). For instance, foliar application of silicon has been reported to enhance rice yield (Vieira et al., 2020; Flores et al., 2022). At the physiological level, foliar silicon supplementation has also been reported to improve photosynthetic apparatus, thereby increasing photosynthetic efficiency as reported by Anand et al. (2018) and Dorairaj et al. (2020). Nevertheless, there is a notable gap in literature since most studies on foliar silicon (Si) application in rice have focused either exclusively on yield-related parameters or solely on physiological responses. In addition, previous studies have generally restricted Si treatments to selected stages of the rice growth cycle, rather than supplying them across all critical phenological stages. Based on this gap, our research hypothesis proposes that foliar application of different liquid Si formulations at the most determining stages of rice development may have different effects on crop productivity and physiological performance. Consequently, the primary objective of this study was to evaluate the effects of different timings and concentrations of foliar applications of two Si-based biostimulants on rice growth, biomass accumulation, grain yield and physiological status. This was achieved through a controlled pot experiment, which incorporated the interactions between two liquid Si formulations, two application concentrations, and foliar treatments across the key phenological stages of rice development.

## Materials and methods

### Plant material and growth conditions

The experiment was carried out in 2023 at the greenhouse facilities of the Faculty of Biology, University of Barcelona, Spain (41°23’6” S, 2°7’12” W). The microclimatic conditions inside the greenhouse were monitored continuously with dataloggers (model Lascar EasyLog EL-USB-2, Lascar Electronics, Wiltshire, UK). Throughout the experimental period, greenhouse temperature was maintained within the range from 25°C to 44°C, with relative humidity kept at approximately 60% (Detailed information is included in **Supplementary Table 1**). Rice (*Oryza sativa* L.) cv. Bomba (Spanish cultivar characterized by high plant size and excellent grain organoleptic properties) was cultivated in pots on a dedicated table equipped with a drip irrigation system. A total of 50 pots were used, with ten pots assigned to each of the five experimental treatments. Each 10 L pot was filled with a substrate mixture composed of acid blonde peatmoss (Floratorf, Oldenburg, Germany) vermiculite, and perlite in a 2:1:1 ratio. Fifteen seeds were directly sown per pot; however, only the five most homogeneous seedlings were maintained before the application of the emergence treatment. No fertilizer was added to the growing substrate. Each pot received a half-strength Hoagland nutrient solution (Hoagland and Arnon, 1938), which was adjusted throughout the experimental period to maintain soil water at field capacity.

### Experimental design and treatments

**Figure 1:**
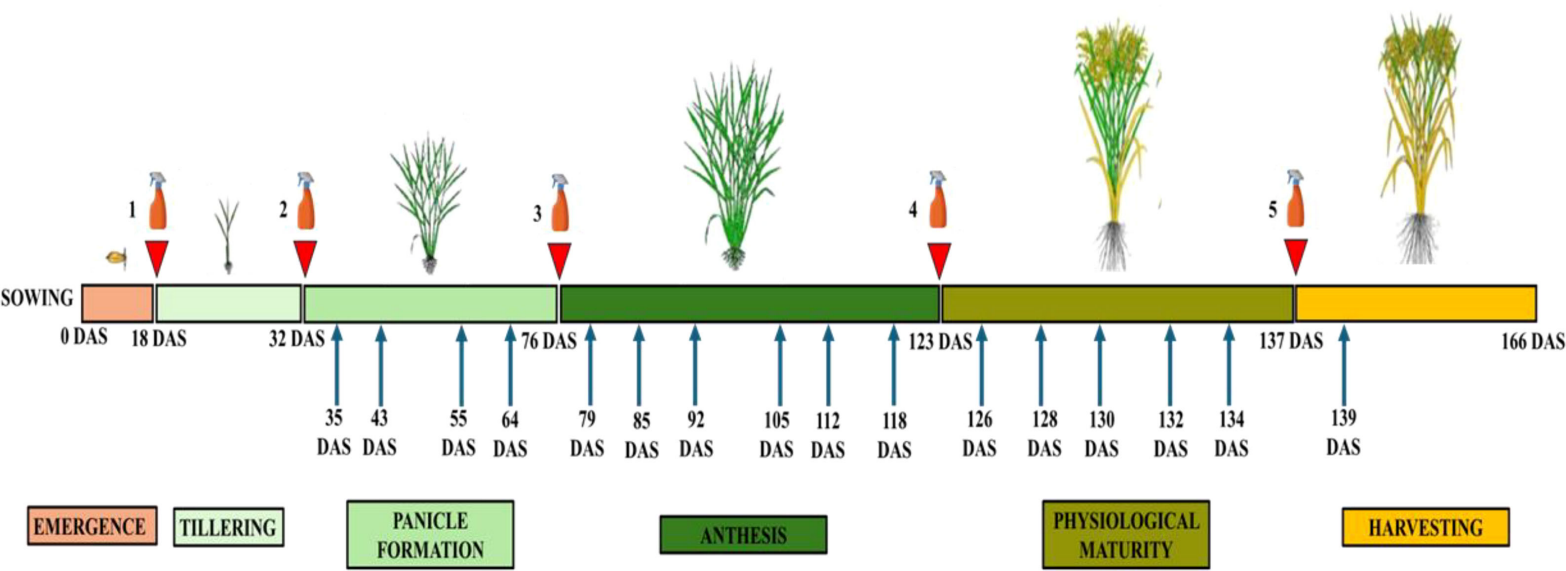
Schematic representation of the rice growth cycle and timing of foliar applications of silicon-based biostimulants. Foliar treatments (indicated by spray icons and red arrowheads) were applied at the beginning of five key developmental stages: tillering, panicle formation, anthesis, physiological maturity and harvesting. Blue arrows represent periodic physiological measurements conducted throughout the experiment. The phenological stages illustrated include emergence, tillering, panicle formation, anthesis, physiological maturity, and harvesting. DAS: indicates days after sowing.

The experimental design consisted of a completely randomized block design with five treatments including 10 replications per treatment. The treatments involved the foliar application of two mineral biostimulant formulations provided by APTUS PLANT TECH (Aptus Holland, IB.ECO BV, Nuth, the Netherlands): F1 (Siliforce-4) and F2 (Simosa). Each biostimulant was tested at two concentrations, 1:1000 (T1) and 1:500 (T2). The F1 formulation consisted of a mixture of elements such as Si (mono-orthosilicic acid, H_4_SiO_4_), Mo, Ca, Zn, and B, while F2 contained only mono-orthosilicic acid (H_4_SiO_4_). A control treatment was included in which distilled water was included in place of the biostimulant solutions. Biostimulants were applied via foliar spray using different sprayer bottles for each formulation to avoid cross-contamination. Applications were conducted during the early morning hours to minimize evaporation losses. All solutions were consistently prepared immediately before application, and their pH was adjusted between 5.5 and 5.8 using NaOH to enhance foliar absorption. Foliar treatments were administered at the beginning of key phenological stages of rice development: tillering, panicle formation, anthesis, physiological maturity and harvesting (see **Figure 1**). No pests or disease management practices were applied during the experiment just to better assess the performance of the biostimulants.

### Biomass and yield parameters

Plant height, tiller number per pot, grain yield, and aerial biomass were assessed at the end of the reproductive stage. Plant height was measured manually using a ruler, from the base of the stem at soil level to the tip of the tallest tiller. Tiller numbers were counted directly for each pot. Following harvest, plants were manually threshed to assess grain yield. The harvested aerial biomass was then dried in a forced-air oven at 70 °C for 48 h to determine the respective dry weight. The harvest index was calculated as the ratio of grain yield to total aerial dry biomass.

### Physiological determinations

Various physiological determinations were carried out at different timepoints corresponding to different developmental stages in rice, including panicle formation, anthesis, physiological maturity and harvesting.

### Vegetation indices

During the experimental growing period, individual zenithal photographs of each pot in the greenhouse were captured using an RGB ILCE-QX1 camera (Sony, Tokio, Japan). Images were taken at different time points until the onset of physiological maturity. Four vegetation indices were derived from the images using Image J’s Cereal Scanner plugin (https://integrativecropecophysiology.com/software-development/cerealscanner/): Normalized Green-Red Difference Index (NGRDI), Triangular Greenness Index (TGI), Green Area (GA) and Greener Area (GGA). The GA value of the images was calculated as the sum of pixels in the hue-saturation-intensity (HIS) color space within a hue range from 60° to 180°, while GGA was computed similarly but within a narrower hue range from 80° to 180°, representing the proportion of more actively green leaf area (Casadesús et al., 2007). The other vegetation indices were calculated using the following equations:

(1) NGRDI=Green DN−Red DNGreen DN+Red DN

(Hunt et al., 2005) (**Equation 1**)

(2) TGI=−0,5 [190 (R670−R550)−120(R670−R480)]

(Hunt et al., 2013) (**Equation 2**)

The Triangular Greenness Index (TGI) was formulated by using a triangular region encompassing the spectral characteristics of chlorophyll. The vertices of the triangle were defined as 670 nm, 550 nm, and 480 nm. This index is a good indicator of the chlorophyll content in leaves (Hunt et al., 2013).

### Leaf pigment and NBI determinations

Leaf pigments were assessed in the flag leaf of rice plants using a portable leaf-clip sensor (Dualex, Dualex Force-A, Orsay, France). The Dualex sensor facilitates non-destructive determinations of chlorophyll (Chl, µg cm^-2^), flavonoid (Fla, dimensionless index), and anthocyanin (Anth, dimensionless index) concentration, analyzing the light transmitted through the leaf at specific wavelengths (λ = 375 nm for flavonoids and λ = 345 nm for chlorophylls) (Cerovic et al., 2012). Additionally, this sensor computes the nitrogen balance index (NBI), representing the Chl/Flav ratio in relation to nitrogen and carbon allocation dynamics (Cerovic et al., 2015). In accordance with experimental protocol, three recently fully expanded (i.e., non-senescent) leaves were measured once per pot. The measurements were performed at different timepoints in the physiological maturity stage.

### Plant photosynthesis and gas exchange parameters

Leaf gas exchange parameters related to photosynthesis and transpiration were measured with a LI-6800 portable photosynthesis system (LI-COR Corporate, Lincoln, USA). Measurements were conducted under controlled conditions: a leaf temperature of 25°C, saturation photosynthetically active radiation (1,500 μmol m^-2^ s^-1^), ambient CO_2_ concentration of 400 ppm, and a relative humidity of 50%. The flag leaves selected for gas exchange measurements had the same morphological characteristics as those used for pigment analysis. The main physiological parameters studied included net photosynthetic rate (A), transpiration rate (E), ambient CO_2_ concentration (ca), intercellular CO_2_ concentration (ci), the ratio of ci to ca (ci/ca), stomatal conductance (gs), leaf vapor pressure deficit (VPD), and intrinsic water use efficiency (WUE) calculated as the A/gs ratio. Measurements were recorded at three different timepoints during the phenological stages of anthesis and physiological maturity: 79, 105 and 126 days after sowing (DAS).

### Total nitrogen and carbon and stable isotope analyses

The leaves selected for elemental analysis and stable isotope signatures underwent to successive washing cycles with both tap and distilled water. Subsequently, they were subjected to desiccation within an oven set at 60 °C for a duration of two days. Finally, the desiccated leaves were finely ground into a powder of uniform consistency. A subsample of the dried leaf powder was used for the determination of total carbon and nitrogen concentrations, as well as the stable isotopic signatures of carbon (^13^C/^12^C ratio) and nitrogen (^15^N/^14^N ratio). The analyses were conducted at the Scientific Facilities of the University of Barcelona. Approximately 1 mg of subsamples were weighed into tin capsules, followed by analyses carried out through an elemental analyzer (Flash 1112 EA; ThermoFinnigan, Schwerte, Germany) integrated with an isotope ratio mass spectrometer (Delta C IRMS, ThermoFinnigan), which operated in continuous flow mode. Primarily standard for carbon isotope analysis was the Pee Dee Belemnite (PDB) calcium carbonate. International isotope secondary standards with established ^13^C/^12^C ratios (IAEA CH7 3, polyethylene foil; IAEA CH6, sucrose; USGS 40, l-glutamic acid) were employed, ensuring an analytical precision of 0.1‰. Nitrogen isotope analysis were performed using as a primary standard N_2_ in air (IAEA-N-2) and as an international isotope secondary standards (IAEA N1, IAEAN2, IAEANO3, and USGS40) with a precision of 0.3‰. Total nitrogen and carbon content in leaves were expressed in percentage (%).

The carbon and nitrogen isotopic compositions, denoted as δ^13^C and δ^15^N respectively, were expressed utilizing the following notation (Coplen, 2008) (**Equation 3**):

(3) $$\delta^{13}\text{C }or\;\delta^{15}\text{N }\left(‰\right)\;=\;\left[\left(\mathrm{Rsample}/\mathrm{Rstandard}\right)-1\right]\times 1000$$

where δ^13^C and δ^15^N represents the ratios of isotopes ^13^C/^12^C and ^15^N/^14^N in the sample, respectively, both expressed in ‰. Meanwhile, R standard denotes the molar abundance ratio of the secondary standard calibrated against the primary standard. Each treatment consisted of 3 replicates.

### Mineral composition of rice grains

To determine the mineral concentration in the rice grain, for each of the three replicates of each treatment, 100 mg of milled material was weighed in Teflon^®^ beakers and digested in 2 mL HNO_3_ and 1 mL H_2_O_2_ at 90°C overnight at the ionomics service of the CEBAS (Centro de Edafología y Biología Aplicada del Segura), Murcia, Spain. Digests were diluted in 30 mL of MilliQ water and refrigerated until analysis. The mineral concentration in the digests was determined by Induced Coupled Plasma Optical Emission Spectrometry (ICP-OES) for calcium (Ca), iron (Fe), potassium (K), magnesium (Mg), sodium (Na), phosphorus (P), sulfur (S), nickel (Ni), and for silicon Si (Optima 3200rl, Perkin Elmer); and by Induced Coupled Plasma Mass Spectrometry (ICP-MS, Elan6000, Perkin Elmer) for boron (B), copper (Cu), zinc (Zn), manganese (Mn), molybdenum (Mo), cobalt (Co), lead (Pb), cadmium (Cd), chromium (Cr), rubidium (Rb), lithium (Li), strontium (Sr), titanium (Ti), thallium (Tl), and bismuth (Bi). Based on these analyses, the maximum daily intake (MDI) per person was determined, and the acceptability of each element was evaluated in relation to the maximum thresholds established by EFSA (2009). The MDI was calculated as the product of the average rice consumption (ARC) per person per day and the maximum concentration recorded (MCR) for each element across the different treatments evaluated in the experiment.

### Statistical analysis

Statistical differences among treatments were evaluated using a one-way analysis of variance (ANOVA), followed by Fisher’s Least Significant Difference (LSD) test at the 5% probability level for mean comparison. Normality and homogeneity of the variances were checked using the Shapiro-Wilk test. All statistical analyses were performed using Statgraphics Centurion XVI (Statpoint Technologies, Inc. Warrenton, VA, USA).

## Results

### Effects of silicon formulations, concentrations, and foliar application timing on biomass and yield parameters

The foliar application of Siliforce-4 and Simosa at two different concentrations did not significantly affect plant height or 1000-grain weight at the end of the experimental period. In terms of tiller number per pot, Siliforce-4 application resulted in a significant decrease compared to the control treatment, whereas Simosa application enhanced the tiller number in rice plants in a dose-dependent manner compared to the control treatment. Similarly, aerial dry weight was significantly increased only in rice plants treated with Simosa, regardless of concentration. Regarding the harvest index, the highest value was observed in rice plants treated with Siliforce-4 at 1:500 dilution, whereas the rest of the treatments showed significantly lower values compared to the control (**Table 1**).

**Table 1:** Effects of foliar applications of Siliforce-4 (F1) and Simosa (F2) at two different concentrations (T1 and T2) on biomass and yield parameters in rice plants over the experimental period.

Treatments	Plant height (cm)	Tiller number pot^-1^	Aerial dry weight (g pot^-1^)	Grain yield (g pot^-1^)	1000-grain weight (g)	Harvest index (-)
CK	112,94 ± 14,63 a	9,84 ± 5,09 b	145,86 ± 61,80 b	45,36 ± 14,54 ab	23,14 ± 1,70 a	0,32 ± 0,04 ab
F1T1 (1:1000)	115,70 ± 18,36 a	7,50 ± 4,02 c	131,14 ± 61,74 b	38,35 ± 20,34 b	24,07 ± 1,30 a	0,29 ± 0,04 bc
F1T2 (1:500)	106,69 ± 19,04 a	7,20 ± 3,99 c	126,63 ± 66,65 b	44,32 ± 24,53 ab	22,43 ± 1,99 a	0,34 ± 0,06 a
F2T1 (1:1000)	125,20 ± 12,80 a	11,38 ± 4,83 ab	198,76 ± 61,05 a	51,10 ± 15,19 ab	22,82 ± 1,18 a	0,26 ± 0,03 c
F2T2 (1:500)	112,22 ± 19,68 a	11,88 ± 4,39 a	222,42 ± 71,61 a	59,09 ± 19,28 a	23,69 ± 2,00 a	0,27 ± 0,05 c

Treatment values are the means ± standard deviation of 10 replicates per treatment. Values with different letters within a column are significantly different at p< 0.05 (analysis of variance and least significant difference test).

### Effects of silicon formulations, concentrations, and foliar application timing on vegetation indices

**Figure 2:**
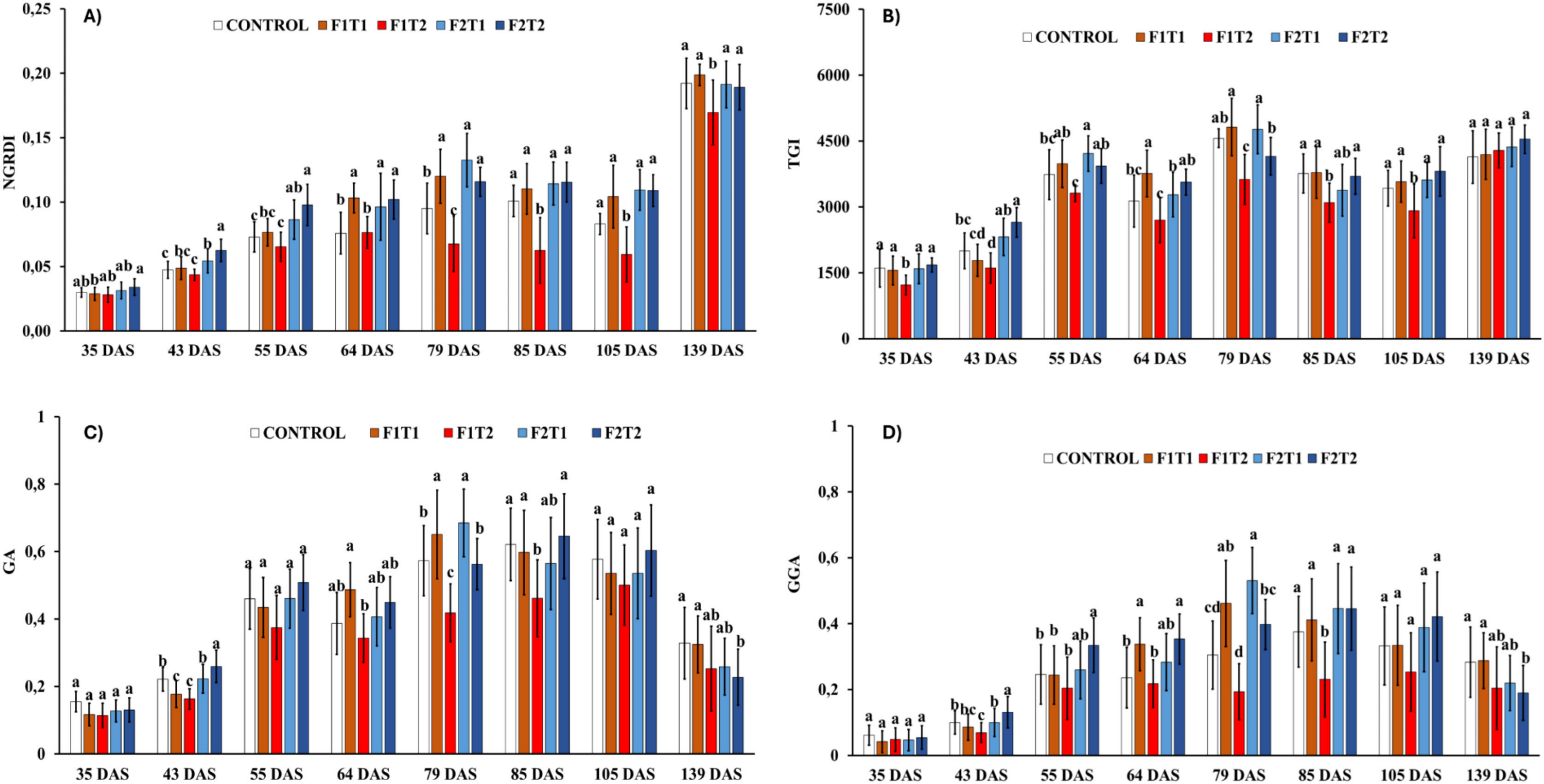
Effects of foliar applications of Siliforce-4 (F1) and Simosa (F2) at two different concentrations (T1 and T2) on vegetation indices: **(A)** Normalized Green-Red Difference Index (NGRDI), **(B)** Triangular Greenness Index (TGI), **(C)** Green Area (GA) and **(D)** Greener Area (GGA) in rice plants at different timepoints during the experimental period. Treatment values are the means ± standard deviation of 10 replicates per treatment. Values with different letters are significantly different at p< 0.05 (analysis of variance and least significant difference test).

The values of Normalized Green-Red Difference Index (NGRDI) increased slightly across the different timepoints measured. However, plants treated with Siliforce-4 at 1:500 dilution consistently showed the lowest NGRDI values, with this trend becoming more accentuated after the third foliar application (79 DAS). The Triangular Greenness Index (TGI) followed a similar temporal pattern to that of NGRDI. Nevertheless, unlike NGRDI, no significant differences in TGI values were observed among treatments after the fifth foliar application (139 DAS). For Green Area (GA) and Greener Area (GGA), a biphasic trend was observed: both indices increased across all treatments until the third foliar application (79 DAS), followed by a gradual decline toward the end of the experimental period. Notably, no consistent differences in GA or GGA values were found between treatments throughout the study (**Figure 2**).

### Effects of silicon formulations, concentrations, and foliar application timing on leaf pigment concentrations

**Figure 3:**
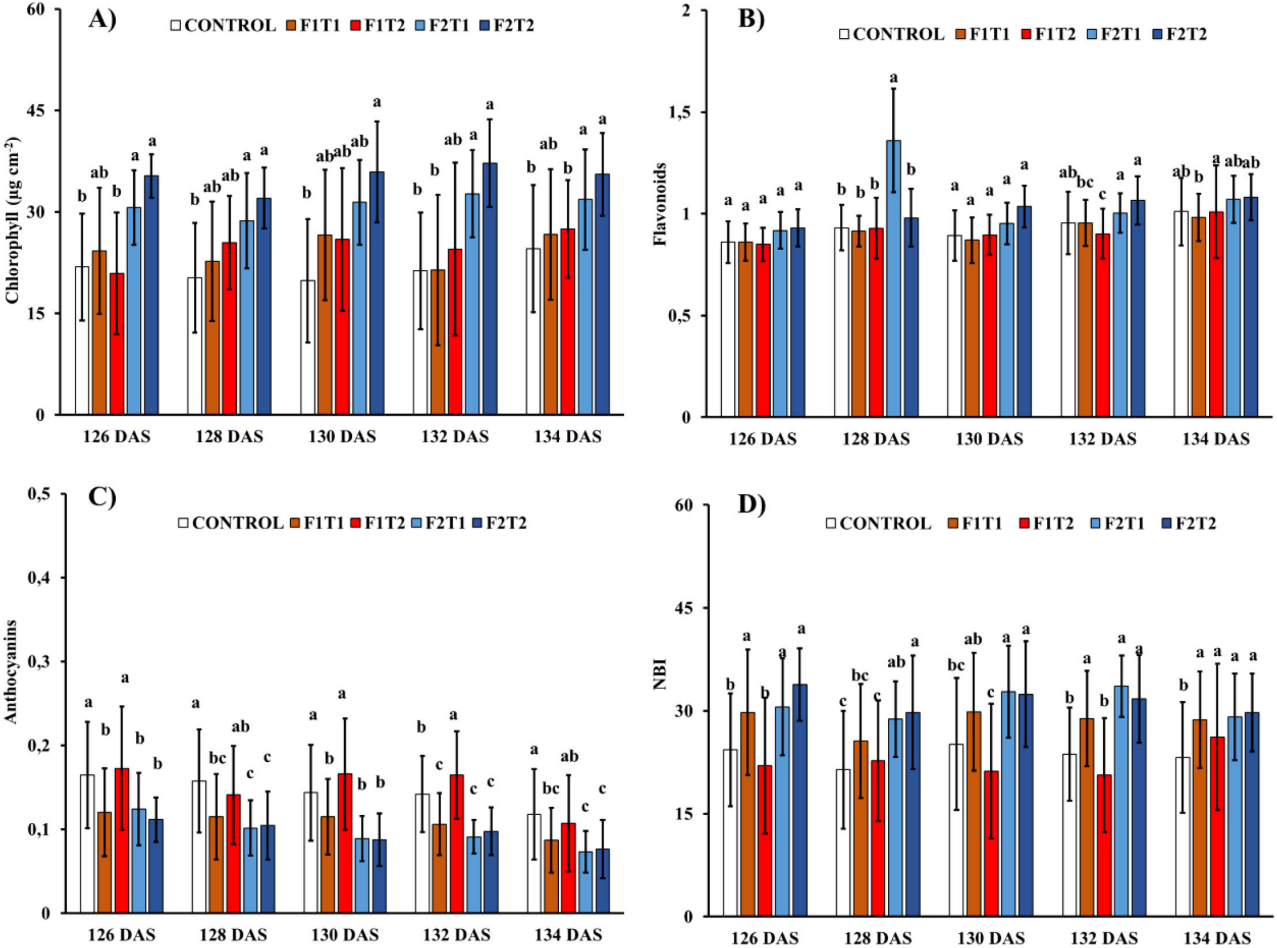
Effects of foliar applications of Siliforce-4 (F1) and Simosa (F2) at two different concentrations (T1 and T2) on leaf pigments in rice plants at different timepoints during the experimental period. **(A)** Chlorophyll, **(B)** Flavonoids, **(C)** Anthocyanins and **(D)** NBI. Treatment values are the means ± standard deviation of 10 replicates per treatment. Values with different letters are significantly different at p< 0.05 (analysis of variance and least significant difference test).

Leaf chlorophyll concentration followed a similar temporal trend across all treatments showing consistently the highest values in plants treated with Simosa regardless of concentration. In contrast, flavonoids concentration did not show consistent patterns either across treatments or over time. Leaf anthocyanin concentration remained relatively stable within treatments throughout the experimental period. Nevertheless, plants treated with Simosa, regardless of concentration, declined leaf anthocyanin concentration, whereas those treated with Siliforce-4 at 1:500 dilution showed the highest concentrations being similar to the control treatment. The Nitrogen Balance Index (NBI), followed a similar pattern to that of anthocyanin concentration in terms of treatment stability over time. Nevertheless, in contrast to anthocyanins, NBI values increased in plants treated with Simosa regardless of concentration. Plants treated with Siliforce-4 at 1:500 dilution showed the highest NBI values, except at the final timepoint (139 DAS), when no significant differences were observed among treatments (**Figure 3**).

### Effects of silicon formulations, concentrations, and foliar application timing on gas exchange parameters

**Figure 4:**
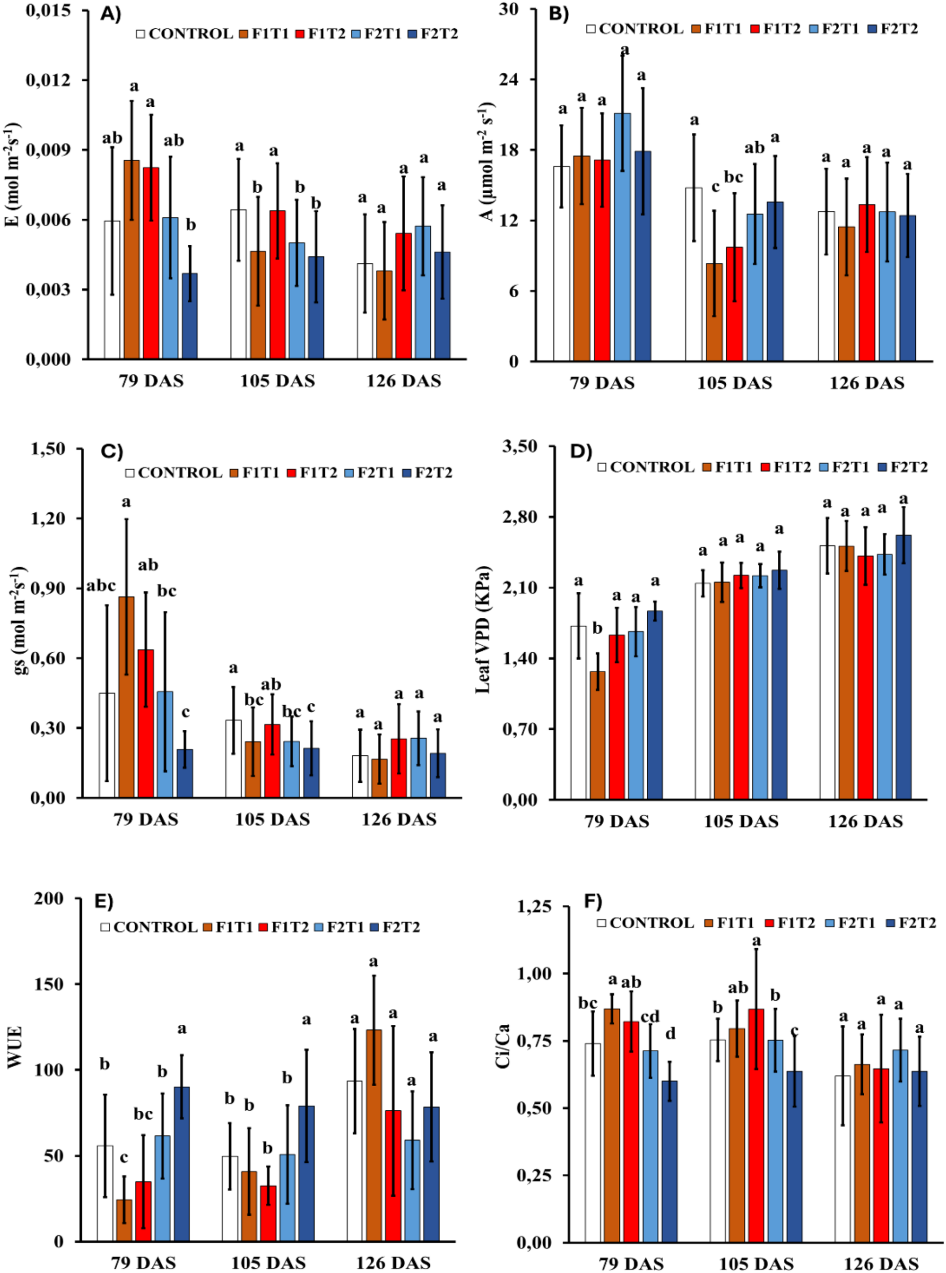
Effects of foliar applications of Siliforce-4 (F1) and Simosa (F2) at two different concentrations (T1 and T2) on gas exchange parameters in rice plants at different timepoints during the experimental period. **(A)** Transpiration rate (E), **(B)** net photosynthetic rate (A), **(C)** stomatal conductance (gs), **(D)** leaf vapor pressure deficit (VPD), **(E)** intrinsic water use efficiency (WUE) and **(F)** the ratio of ci to ca (ci/ca). Treatment values are the means ± standard deviation of 10 replicates per treatment. Values with different letters within a column are significantly different at p< 0.05 (analysis of variance and least significant difference test).

Net photosynthetic rate (A) and transpiration rate (E) showed similar patterns across treatments and timepoints, without clear differences. Stomatal conductance (gs) showed a general decreasing trend over time; however, no consistent differences were observed among treatments. Leaf vapor pressure deficit (VPD) showed similar values between treatments and across all time points. Regarding water use efficiency, treatments showed a similar trend at the first two timepoints, with the highest WUE observed in rice plants treated with Simosa at 1:500 dilution. However, by the final timepoint, no significant differences in WUE were observed among treatments. For the ratio ci/ca, there were no clear patterns across treatments at the initial timepoints, and no significant differences were observed at the final measurement (**Figure 4**).

### Effects of silicon formulations, concentrations, and foliar application timing on nitrogen and carbon isotopes determination

A significant decline in carbon isotope composition was observed in rice plants treated with Simosa, regardless of concentration, while nitrogen isotope composition, and total nitrogen and carbon leaf concentration remained without changes under the different treatment tested at the end of the experimental period (**Table 2**).

**Table 2:** Effects of foliar applications of Siliforce-4 (F1) and Simosa (F2) at two different concentrations (T1 and T2) on nitrogen and carbon isotopes determination in rice plants.

	N concentration (%)	δ^15^N (‰)	C concentration (%)	δ^13^C (‰)
CK	2,02 ± 0,04 a	2,03 ± 0,34 a	39,63 ± 1,37 a	-30,25 ± 0,31 a
F1T1 (1:1000)	1,88 ± 0,07 a	2,36 ± 0,35 a	40,08 ± 1,05 a	-29,62 ± 0,32 a
F1T2 (1:500)	1,49 ± 0,28 a	1,66 ± 0,21 a	29,56 ± 6,60 a	-29,88 ± 0,14 a
F2T1 (1:1000)	1,93 ± 0,24 a	2,59 ± 0,26 a	40,63 ± 0,48 a	-28,35 ± 0,49 b
F2T2 (1:500)	1,55 ± 0,67 a	2,28 ± 0,54 a	32,31 ± 12,49 a	-28,86 ± 0,47 b

Treatment values are the means ± standard deviation of 3 replicates per treatment. Values with different letters within a column are significantly different at p< 0.05 (analysis of variance and least significant difference test).

### Effects of silicon formulations, concentrations, and foliar application timing on the mineral composition of rice grains

Phosphorus (P), calcium (Ca), and sodium (Na) concentrations in rice grains remained without changes under the different treatments applied. In contrast, sulfur (S) and potassium (K) concentration showed a similar response pattern, both showing their highest value in plants treated with Siliforce-4 at 1:500 dilution, while a significant decline was observed in grains from plants treated with Simosa, regardless of concentration. Regarding K, rice plants treated with Siliforce-4 at 1:500 dilution showed the highest concentration in grains (**Table 3**).

**Table 3:** Effects of foliar applications of Siliforce-4 (F1) and Simosa (F2) at two different concentrations (T1 and T2) on macronutrients concentrations (expressed in g 100 g^-1^) in rice grains.

	P	S	K	Ca	Mg	Na
CK	0,143 ± 0,022 a	0,139 ± 0,004 ab	0,62 ± 0,08 ab	0,086 ± 0,005 a	0,152 ± 0,005 ab	0,63 ± 0,06 a
F1T1 (1:1000)	0,146 ± 0,010 a	0,129 ± 0,008 bc	0,53 ± 0,03 ab	0,091 ± 0,011 a	0,141 ± 0,005 bc	0,53 ± 0,03 a
F1T2 (1:500)	0,156 ± 0,009 a	0,148 ± 0,012 a	0,65 ± 0,12 a	0,087 ± 0,007 a	0,160 ± 0,004 a	0,65 ± 0,13 a
F2T1 (1:1000)	0,137 ± 0,001 a	0,122 ± 0,005 c	0,54 ± 0,06 ab	0,087 ± 0,021 a	0,131 ± 0,011 c	0,53 ± 0,05 a
F2T2 (1:500)	0,138 ± 0,012 a	0,125 ± 0,005 c	0,52 ± 0,02 b	0,090 ± 0,012 a	0,137 ± 0,008 c	0,52 ± 0,02 a

Treatment values are the means ± standard deviation of 3 replicates per treatment. Values with different letters within a column are significantly different at p< 0.05 (analysis of variance and least significant difference test).

Boron (B) and iron (Fe) concentrations in rice grains did not show consistent patterns across the different treatments. In contrast, copper (Cu) and zinc (Zn) concentrations significantly declined in plants treated with Simosa. Nickel (Ni) concentration remained without changes regardless of treatment. Molybdenum (Mo) and cobalt (Co) concentrations followed a similar pattern, with the highest values observed in grains from plants treated with Simosa at 1:500 dilution. For manganese (Mn) and lead (Pb), the highest concentrations were recorded in grains from plants treated with Siliforce-4 at 1:500 dilution (**Table 4**).

**Table 4:** Effects of foliar applications of Siliforce-4 (F1) and Simosa (F2) at two different concentrations (T1 and T2) on micronutrients and heavy metal concentrations (expressed in mg Kg^-1^) in rice grains.

	B	Cu	Fe	Zn	Mn	Mo	Co	Ni	Pb
CK	7,93 ± 1,04 ab	11,65 ± 0,49 ab	22,49 ± 2,56 ab	56,63 ± 1,32 ab	10,05 ± 2,29 b	0,57 ± 0,16 b	0,21 ± 0,06 b	0,62 ± 0,04 a	0,12 ± 0,04 b
F1T1	6,60 ± 1,13 b	10,28 ± 1,63 bc	27,51 ± 11,68 ab	51,23 ± 6,17 bc	9,94 ± 1,04 b	0,65 ± 0,06 b	0,14 ± 0,01 b	0,68 ± 0,13 a	0,24 ± 0,10 ab
F1T2	8,42 ± 0,83 ab	12,80 ± 1,68 a	30,49 ± 6,04 a	62,73 ± 0,93 a	13,16 ± 1,19 a	0,74 ± 0,08 b	0,36 ± 0,04 b	0,59 ± 0,01 a	0,36 ± 0,08 a
F2T1	7,91 ± 1,52 ab	7,84 ± 0,40 d	14,60 ± 1,56 b	47,52 ± 1,49 c	8,29 ± 0,43 b	0,53 ± 0,10 b	0,45 ± 0,02 b	0,60 ± 0,08 a	0,29 ± 0,19 ab
F2T2	9,56 ± 1,27 a	8,54 ± 1,11 cd	18,80 ± 1,80 ab	47,48 ± 4,66 c	8,43 ± 1,55 b	2,42 ± 0,93 a	1,43 ± 0,83 a	0,80 ± 0,34 a	0,28 ± 0,13 ab

	Cd	Cr	Rb	Si	Li	Sr	Ti	Tl	Bi
CK	0,041 ± 0,006 ab	0,45 ± 0,07 a	2,00 ± 0,35 b	0,58 ± 0,17 a	0,27 ± 0,09 b	1,61 ± 0,08 b	0,63 ± 0,26 a	0,81 ± 0,09 a	0,28 ± 0,10 b
F1T1	0,028 ± 0,009 ab	0,54 ± 0,20 a	2,11 ± 0,26 ab	0,64 ± 0,05 a	0,24 ± 0,08 b	1,84 ± 0,34 b	1,07 ± 0,51 a	0,78 ± 0,14 a	0,23 ± 0,16 b
F1T2	0,065 ± 0,016 a	0,58 ± 0,28 a	2,04 ± 0,19 ab	0,70 ± 0,12 a	0,34 ± 0,03 ab	1,57 ± 0,36 b	0,71 ± 0,42 a	0,78 ± 0,10 a	0,36 ± 0,04 b
F2T1	0,011 ± 0,001 b	0,43 ± 0,25 a	2,93 ± 0,58 a	0,48 ± 0,06 a	0,23 ± 0,07 b	2,12 ± 0,48 ab	0,31 ± 0,06 a	0,74 ± 0,05 a	0,35 ± 0,16 b
F2T2	0,071 ± 0,057 a	0,42 ± 0,10 a	2,88 ± 0,83 ab	0,72 ± 0,13 a	0,58 ± 0,31 a	2,61 ± 0,59 a	1,05 ± 0,30 a	0,82 ± 0,15 a	1,43 ± 0,83 a

Treatment values are the means ± standard deviation of 3 replicates per treatment. Values with different letters within a column are significantly different at p< 0.05 (analysis of variance and least significant difference test).

Cadmium concentration in rice grains did not show consistent patterns across the different treatments. Chromium (Cr), silicon (Si), titanium (Ti) and thallium (Tl) concentrations remained without changes regardless of treatment. Lithium (Li), strontium (Sr) and bismuth (Bi) concentrations followed a similar pattern, with the highest value observed in grains from plants treated with Simosa at 1:500 dilution. For rubidium (Rb), the highest concentration was recorded in grains from plants treated with Siliforce-4 at 1:1000 dilution (**Table 4**).

## Discussion

### Effects of silicon foliar application on biomass and yield parameters

Silicon application has been reported to exert a positive influence on plant growth and yield promoting effective allocation of assimilates toward reproductive development (Bhardwaj et al., 2023). In our experiment, regarding yield parameters assessed, only the foliar application of Simosa enhanced significantly the tiller number, aerial dry weight and grain yield in a dose-dependent manner compared to the control treatment. Nevertheless, an opposite trend was observed for the harvest index. Similar results have been reported by other researchers under similar conditions (Jawahar et al., 2015; Cuong et al., 2017; Kheyri et al., 2019). The sequential foliar application of two different chemical fertilizers (Siliforce-4 and Simosa) over the experimental period did not result in significant enhancements in either plant height or 1000-grain weight in rice plants compared to the control treatment. Nevertheless, findings reported about the effect of silicon foliar application on rice plant height in the existing literature remain inconsistent. For instance, Berahim et al. (2021) have reported significant declines in plant height under similar conditions, while other studies reported a clear enhancement of plant height in rice plants under the application of Si (Ahmad et al., 2013; Mohamed et al., 2015). Enhanced plant height can be attributed to improved erectness of leaves and stems due to Si deposition in the cell wall, which reduces mutual shading under high plant density (Cuong et al., 2017). The differential responses between the two fertilizers in our experiment may be attributed to variations in their chemical composition, as well as the presence of adjuvants which can influence the uptake efficiency of the biostimulant. These results highlight the importance of informed product selection, highlighting that optimized formulations can enhance product uptake efficiency and, consequently, result in better agronomic performance.

### Effects of silicon foliar application on vegetation indices

Vegetation indices are widely recognized as reliable, non-destructive indicators of plant physiological status and stress responses (Ye et al., 2023; Aman et al., 2025). In our experiment, both the NGRDI and TGI showed similar trends, showing slight increase across the different sampling periods but without clear differences among silicon treatments. Given that these indices are strongly associated with canopy greenness, our results indicated that the sequential foliar application of silicon may contribute to maintaining higher canopy photosynthetic potential and vigor during critical growth stages, as previously reported (Lavinsky et al., 2016). Similarly, the green area (GA) and greener area (GGA) displayed a similar trend: both increased steadily until the third foliar application and subsequently declined toward the end of the experimental period. Although no consistent treatment-specific differences were noted, the gradual increase can be linked with the positive role of silicon in improving leaf erectness and enhancing light interception, thereby promoting canopy expansion, efficiency, and uniform greenness distribution across the crop stand (Ma, 2003; Xu et al., 2023). The subsequent decline is likely attributable to natural crop senescence and the progressive reallocation of assimilates from vegetative tissues to reproductive organs to ensure grain filling and yield formation (Lee and Masclaux-Daubresse, 2021; Mohapatra and Sahu, 2021).

### Effects of silicon foliar application on pigment concentrations

In crop plants, elevated chlorophyll concentrations are indicative of adequate plant health and nutrient status, whereas increased levels of flavonoids and anthocyanins generally reflect the activation of stress-response pathways under adverse conditions (Pavlović et al., 2014; Yan et al., 2022). In our experiment, the application of foliar silicon products resulted in a significant increase in chlorophyll concentrations across the different sampling periods, with rice plants treated with Simosa showing the highest values. Similar trends have been reported in previous studies, where the application of silicon compounds enhanced chlorophyll concentrations (Elshayb et al., 2021; El-Okkiah et al., 2022; Abd-El-Aty et al., 2024). This increase in pigment concentration can be attributed to the positive role of foliar application of silicon in improving the photosynthetic efficiency by enhancing the chlorophyll stability through the reduction of oxidative damage, strengthening leaf structures, and delaying senescence (Ramírez-Olvera et al., 2021). In contrast, leaf flavonoid concentrations did not show consistent patterns across treatments or sampling times. This phenomenon can be explained by the enhanced antioxidant potential induced by foliar silicon application, as previously demonstrated (Elshayb et al., 2021; Chen et al., 2024), combined with the favorable growth conditions maintained in our experimental design, which likely minimized stress-induced fluctuations. Anthocyanin accumulation, often triggered by stress signals such as oxidative imbalance, nutrient deficiency, or senescence (Naing and Kim, 2021; Jan et al., 2024) was generally lower in silicon-treated plants. An exception was reported with Siliforce-4 at 1:500 dilution, where higher anthocyanin levels were recorded. Overall, the reduced anthocyanin accumulation in silicon treatments supports the notion of improved cellular redox balance, consistent with the trends observed in flavonoid concentrations. This aligns with the widely reported role of silicon in alleviating oxidative stress through the suppression of anthocyanin accumulation (Kim et al., 2017; Mostofa et al., 2021). Furthermore, the nitrogen balance index showed a significant increase in plants treated with Simosa compared to the control treatment across all recorded time points. These results indicated a more efficient nitrogen allocation towards photosynthetic potential, reflected in increased chlorophyll concentrations, rather than diversion of nitrogen towards stress-related metabolites such as flavonoids (Cerovic et al., 2015).

### Effects of silicon foliar application on nitrogen concentration and carbon isotope composition

The analysis of carbon and nitrogen isotopes, along with their respective concentrations in leaves, provides reliable indicators of photosynthetic performance, assimilate partitioning, and the uptake, assimilation, and distribution of nitrogen in plants (Brüggemann et al., 2011; Kalcsits et al., 2014). Our analyses revealed only a clear reduction in the carbon isotope composition in leaves of rice plants subjected to foliar application of Simosa, irrespective of the applied concentration, at the end of the experimental period. This reduction in δ^13^C is likely associated with lowered stomatal conductance induced by foliar silicon application, as previously reported Detmann et al. (2012) and Lavinsky et al. (2016). In contrast, no significant variations in total nitrogen and carbon concentration as well as in carbon isotopes were detected in our experiment. This lack of variability can be attributed to the favorable climatic conditions, adequate irrigation at field capacity, and the continuous supply of a standard nutrient solution throughout the experimental period, which collectively ensured plant growth across treatments.

### Effects of silicon foliar application on gas exchange parameters

In our experiment, the net photosynthetic rate (A) and transpiration rate (E) remained similar across treatments and sampling timepoints, without clear differences detected. Stomatal conductance (gs) showed a similar trend, with a decline over time regardless of treatment. Because both A and E are strongly regulated by gs, these parallel dynamics suggest that stomatal behavior was the primary determinant of gas exchange under the conditions of this experiment (Dow et al., 2014; Harrison et al., 2020). The temporal decline in gs may reflect leaf developmental stage, progressive acclimation, or shared environmental influences such as increasing vapor pressure deficit or natural diurnal dynamics, rather than treatment specific effects (Grossiord et al., 2020; López et al., 2021). The lack of variations among treatments further suggest that the experimental conditions were not sufficiently stressful to elicit differential stomatal responses. The stability of ca confirms that the external CO_2_ level was uniform, while the consistency of ci indicates that foliar silicon application did not alter the mesophyll capacity for CO_2_ assimilation (Detmann et al., 2012; Ulloa et al., 2021). Likewise, the lack of clear trends in ci/ca ratios reflects the uniformity of ca and ci measurements. Similarly, leaf VPD did not differ across treatments or sampling periods, indicating that environmental conditions remained stable and reinforcing that observed responses in transpiration and stomatal conductance were intrinsic to plant physiology rather than externally imposed. Finally, water use efficiency displayed higher variability among treatments and showed a slight trend to increase over time. This pattern can be linked to the increase in leaf VPD, which likely contributed to the consequent decrease in gs, while A remained stable throughout the experiment (Grossiord et al., 2020).

### Effects of silicon foliar application on the mineral composition of rice grains

The mineral composition of rice grain is strongly influenced by the availability of soil nutrients during crop growth (Shrestha et al., 2020). In our experiment, the chemical composition of rice grains including macronutrients, micronutrients and heavy metals concentrations was evaluated under the different foliar silicon treatments. There were different trends in the chemical compounds analyzed: while some elements remained unchanged, others increased or decreased in comparison to the control treatment, even within the same chemical group. Grain composition is of particular relevance from both nutritional and toxicological perspectives (Rohman et al., 2014). To assess potential dietary implications, we estimated the daily intake based on an average rice consumption of 100 g dry weight per person and day (Batres-Marquez et al., 2009). Using the concentrations recorded in our experiment, we compared macronutrient and heavy metals levels against the recommended dietary allowances (RDA) and upper thresholds established by EFSA (2009). The results indicated that consumption of the rice produced under these treatments would not exceed the maximum permissible levels for any element, except for copper, which surpassed the threshold by approximately 40% (**Supplementary Table 2**). The elevated copper levels may be associated with specific components of the biostimulant formulation or with an enhanced uptake of this element resulting from the foliar application of the product (Bulgari et al., 2015). Further research should address the bioavailability of copper and the potential effects of milling on its final concentration. This finding is particularly relevant, as excessive copper intake has been associated with adverse health outcomes, including cardiovascular disease, cognitive decline, and cancer (Bost et al., 2016).

## Conclusion

The results of this study demonstrate that foliar silicon application exerted selective and product-dependent effects in rice plants. The foliar application of Simosa enhanced the tiller number, aerial dry weight and grain yield, chlorophyll concentration and nitrogen balance index, thereby promoting resource allocation toward reproductive development. These responses were associated with lower δ^13^C values, consistent with lowered stomatal conductance and higher vegetation indices which are indicative of higher biomass. Nevertheless, no consistent dose-dependent effects were observed, which can be ascribed to limitations in foliar absorption, which may rapidly saturate and restrict further uptake of Si available, Si polymerization, or formulation stability which may decouple applied dose from physiological efficacy. The fact that only Simosa produced clear positive effects highlights that not all silicon formulations are equally effective under the tested conditions. Regarding rice consumption, only copper exceeded the threshold established by EFSA (2009). Although this experiment elucidated rice biomass and physiological responses to foliar silicon, future work should refine dose–response relationships and test findings under field conditions across different environments, to validate agronomic benefits of different silicon formulations.

## Data Availability

The original contributions presented in the study are included in the article/**Supplementary Material**. Further inquiries can be directed to the corresponding author.

## References

[B1] Abd-El-Aty M. S. Kamara M. M. Elgamal W. H. Mesbah M. I. Abomarzoka E. A. Alwutayd K. M. . (2024). Exogenous application of nano-silicon, potassium sulfate, or proline enhances physiological parameters, antioxidant enzyme activities, and agronomic traits of diverse rice genotypes under water deficit conditions. Heliyon 10, e26077. doi: 10.1016/j.heliyon.2024.e26077, PMID: 38434411 PMC10907525

[B2] Ahmad A. Afzal M. Ahmad A. U. H. Tahir M. (2013). Effect of foliar application of silicon on yield and quality of rice (*Oryza sativa*, L.). Cerc. Agro. In Moldova 155, 21–28. doi: 10.2478/v10298-012-0089-3

[B3] Aman M. Khan Z. U. Khan J. Mashori A. S. Ali A. Jabeen N. . (2025). A comprehensive review on crop stress detection: Destructive, non-destructive, and ml-based approaches. Front. Plant Sci. 16. doi: 10.3389/fpls.2025.1638675, PMID: 40978773 PMC12447170

[B4] Anand L. Sreekanth B. Jayalalitha K. Jyothula D. P. B. (2018). Effect of silicon nutrition on photosynthetic attributes of rice (*Oryza sativa* L). Andhra Agric. J. 65, 908–912. doi: 10.20546/ijcmas.2018.711.099

[B5] Araus J. L. Villegas D. Aparicio N. Del Moral L. G. El Hani S. Rharrabti Y. . (2003). Environmental factors determining carbon isotope discrimination and yield in durum wheat under Mediterranean conditions. Crop Sci. 43, 170–180. doi: 10.2135/cropsci2003.1700

[B6] Batres-Marquez S. P. Jensen H. H. Upton J. (2009). Rice consumption in the United States: recent evidence from food consumption surveys. J. Amer. Diet. Assoc. 109, 1719–1727. doi: 10.1016/j.jada.2009.07.010, PMID: 19782171

[B7] Berahim Z. Omar M. H. Zakaria N. I. Ismail M. R. Rosle R. Roslin N. A. . (2021). Silicon improves yield performance by enhancement in physiological responses, crop imagery, and leaf and culm sheath morphology in new rice line, PadiU Putra. BioMed. Res. Int. 1, 6679787. doi: 10.1155/2021/6679787, PMID: 34159198 PMC8187073

[B8] Bhardwaj S. Sharma D. Singh S. Ramamurthy P. C. Verma T. Pujari M. . (2023). Physiological and molecular insights into the role of silicon in improving plant performance under abiotic stresses. Plant Soil 486, 25–43. doi: 10.1007/s11104-022-05395-4

[B9] Bost M. Houdart S. Oberli M. Kalonji E. Huneau J. F. Margaritis I. (2016). Dietary copper and human health: Current evidence and unresolved issues. J. Trace Elem. Med. Biol. 35, 107–115. doi: 10.1016/j.jtemb.2016.02.006, PMID: 27049134

[B10] Brüggemann N. Gessler A. Kayler Z. Keel S. G. Badeck F. Barthel M. . (2011). Carbon allocation and carbon isotope fluxes in the plant-soil-atmosphere continuum: a review. Biogeosciences 8, 3457–3489. doi: 10.5194/bg-8-3457-2011

[B11] Bulgari R. Cocetta G. Trivellini A. Vernieri P. Ferrante A. (2015). Biostimulants and crop responses: a review. Biol. Agric. Hortic. 31, 1–17. doi: 10.1080/01448765.2014.964649

[B12] Casadesús J. Kaya Y. Bort J. Nachit M. M. Araus J. L. Amor S. . (2007). Using vegetation indices derived from conventional digital cameras as selection for wheat breeding in water-limited environments. Ann. Appl. Biol. 150, 227–236. doi: 10.1111/j.1744-7348.2007.00116.x

[B13] Cerovic Z. G. Ghozlen N. B. Milhade C. Obert M. Debuisson S. Le Moigne M. (2015). Nondestructive diagnostic test for nitrogen nutrition of grapevine (*Vitis vinifera* L.) based on dualex leaf-clip measurements in the field. J. Agric. Food Chem. 63, 3669–3680. doi: 10.1021/acs.jafc.5b00304, PMID: 25801210

[B14] Cerovic Z. G. Masdoumier G. Ghozlen N. B. Latouche G. (2012). A new optical leaf clip meter for simultaneous non-destructive assessment of leaf chlorophyll and epidermal flavonoids. Physiol. Plant 146, 251–260. doi: 10.1111/j.1399-3054.2012.01639.x, PMID: 22568678 PMC3666089

[B15] Chack S. Tiwari R. K. Sonia H. Doruk K. Husain R. Singh T. (2023). The silicon: The role on growth and yield of cereals under different abiotic stresses. Pharma Innov. 12, 4285–4289.

[B16] Chan-In P. Jamjod S. Prom-U-Thai C. Rerkasem B. Russell J. Pusadee T. (2024). Application of silicon influencing grain yield and some grain quality features in thai fragrant rice. Plants 13, 1336. doi: 10.3390/plants13101336, PMID: 38794407 PMC11125221

[B17] Chen H. Huang X. Chen H. Zhang S. Fan C. Fu T. . (2024). Effect of silicon spraying on rice photosynthesis and antioxidant defense system on cadmium accumulation. Sci. Rep. 14, 15265. doi: 10.1038/s41598-024-66204-9, PMID: 38961133 PMC11222525

[B18] Coplen T. B. (2008). “ Explanatory Glossary of Terms used in expression of Relative Isotope ratios and gas ratios. IUPAC Provisional Recommendations. Inorganic Chemistry Division,” in Commission on Isotopic Abundances and Atomic Weights. Available online at: http://old.iupac.org/reports/provisional/abstract08/coplen310508.html/.

[B19] Cuong T. X. Ullah H. Datta A. Hanh T. C. (2017). Effects of silicon-based fertilizer on growth, yield and nutrient uptake of rice in tropical zone of Vietnam. Rice Sci. 24, 283–290. doi: 10.1016/j.rsci.2017.06.002

[B20] Detmann K. C. Araújo W. L. Martins S. C. Sanglard L. M. Reis J. V. Detmann E. . (2012). Silicon nutrition increases grain yield, which, in turn, exerts a feed-forward stimulation of photosynthetic rates via enhanced mesophyll conductance and alters primary metabolism in rice. New Phytol. 196, 752–762. doi: 10.1111/j.1469-8137.2012.04299.x, PMID: 22994889

[B21] Deus A. C. F. de Mello Prado R. de Cássia Félix Alvarez R. de Oliveira R. L. L. Felisberto G. (2020). Role of silicon and salicylic acid in the mitigation of nitrogen deficiency stress in rice plants. Silicon 12, 997–1005. doi: 10.1007/s12633-019-00195-5

[B22] Dong Y. Bi X. Wu R. Belfield E. J. Harberd N. P. Christensen B. T. . (2022). The potential of stable carbon and nitrogen isotope analysis of foxtail and broomcorn millets for investigating ancient farming systems. Front. Plant Sci. 13. doi: 10.3389/fpls.2022.1018312, PMID: 36340416 PMC9627502

[B23] Dorairaj D. Ismail M. R. Sinniah U. R. Tan K. B. (2020). Silicon mediated improvement in agronomic traits, physiological parameters and fiber content in *Oryza sativa*. Acta Physiol. Plant 42, 38. doi: 10.1007/s11738-020-3024-5

[B24] Dow G. J. Bergmann D. C. Berry J. A. (2014). An integrated model of stomatal development and leaf physiology. New Phytol. 201, 1218–1226. doi: 10.1111/nph.12608, PMID: 24251982

[B25] EFSA (2009) ( European Food Safety Authority).

[B26] El-Okkiah S. A. El-Afry M. M. Shehab Eldeen S. A. El-Tahan A. M. Ibrahim O. M. Negm M. M. . (2022). Foliar spray of silica improved water stress tolerance in rice (*Oryza sativa* L.) cultivars. Front. Plant Sci. 13. doi: 10.3389/fpls.2022.935090, PMID: 36466243 PMC9709440

[B27] Elshayb O. M. Nada A. M. Ibrahim H. M. Amin H. E. Atta A. M. (2021). Application of silica nanoparticles for improving growth, yield, and enzymatic antioxidant for the hybrid rice ehr1 growing under water regime conditions. Materials 14, 1150. doi: 10.3390/ma14051150, PMID: 33671062 PMC7957767

[B28] E.U (2019). Regulation of the European parliament and of the council laying down rules on the making available on the market of EU fertilising products and amending regulations (EC) no 1069/2009 and (EC) no 1107/2009 and repealing regulation (EC) no 2003/2003. 2019. Available online at: https://eur-lex.europa.eu/legal-content/EN/TXT/?uri=OJ:L:2019:170:TOC. (Access February 02, 2025)

[B29] Farquhar G. D. Ehleringer J. R. Hubick K. T. (1989). Carbon isotope discrimination and photosynthesis. Ann. Rev. Plant Physiol. Plant Mol. Biol. 40, 503–537. doi: 10.1040-2519/89/0601-503502.00

[B30] Farquhar G. D. Richards R. A. (1984). Isotopic composition of plant carbon correlates with water-use efficiency of wheat genotypes. Funct. Plant Biol. 11, 539–552. doi: 10.1071/PP9840539

[B31] Flores R. A. Pessoa-de-Souza M. A. de Andrade A. F. Bueno A. M. de Oliveira Abdala K. de Souza Júnior J. P. . (2022). Does foliar application of silicon under natural water stress conditions increase rice yield in subtropical dry regions? Silicon 14, 3591–3600. doi: 10.1007/s12633-021-01109-0

[B32] Garcia-Caparros P. Ciriello M. Rouphael Y. Giordano M. (2025). The role of organic extracts and inorganic compounds as alleviators of drought stress in plants. Horticulturae 11, 91. doi: 10.3390/horticulturae11010091

[B33] Gracia-Romero A. Kefauver S. C. Fernandez-Gallego J. A. Vergara-Díaz O. Araus J. L. (2019). UAV and ground image-based phenotyping: a proof of concept with durum wheat. Remote Sens. 11, 1–25. doi: 10.3390/rs11101244

[B34] Grossiord C. Buckley T. N. Cernusak L. A. Novick K. A. Poulter B. Siegwolf R. T. . (2020). Plant responses to rising vapor pressure deficit. New Phytol. 226, 1550–1566. doi: 10.1111/nph.16485, PMID: 32064613

[B35] Harrison E. L. Arce Cubas L. Gray J. E. Hepworth C. (2020). The influence of stomatal morphology and distribution on photosynthetic gas exchange. Plant J. 101, 768–779. doi: 10.1111/tpj.14560, PMID: 31583771 PMC7065165

[B36] Hoagland D. R. Arnon D. I. (1938). The water culture method for growing plants without soil. California Agric. Experiment Station Circ. 347, 32.

[B37] Hunt E. R. Cavigelli M. Daughtry C. S. Mcmurtrey J. E. Walthall C. L. (2005). Evaluation of digital photography from model aircraft for remote sensing of crop biomass & nitrogen status. Prec. Agric. 6, 359–378. doi: 10.1007/s11119-005-2324-5

[B38] Hunt E. R. Doraiswamy P. C. McMurtrey J. E. Daughtry C. S. Perry E. M. Akhmedov B. (2013). A visible band index for remote sensing leaf chlorophyll content at the canopy scale. Int. J. Appl. Earth Obser. Geoinfor. 21, 103–112. doi: 10.1016/j.jag.2012.07.020

[B39] Jan R. Asif S. Asaf S. Lubna Khan Z. Kim K. M. (2024). Unveiling the protective role of anthocyanin in rice: insights into drought-induced oxidative stress and metabolic regulation. Front. Plant Sci. 15. doi: 10.3389/fpls.2024.1397817, PMID: 38863532 PMC11165195

[B40] Jawahar S. Vijayakumar D. Bommera R. Jain N. (2015). Effect of silixol granules on growth and yield of rice. Int. J. Curr. Res. Acad. Rev. 3, 168–174.

[B41] Jiang H. Song Z. Su Q. W. Wei Z. H. Li W. C. Jiang Z. X. . (2022). Transcriptomic and metabolomic reveals silicon enhances adaptation of rice under dry cultivation by improving flavonoid biosynthesis, osmoregulation, and photosynthesis. Front. Plant Sci. 13. doi: 10.3389/fpls.2022.967537, PMID: 35991391 PMC9386530

[B42] Jin W. Li L. He W. Wei Z. (2024). Application of silica nanoparticles improved the growth, yield, and grain quality of two salt-tolerant rice varieties under saline irrigation. Plants 13, 2452. doi: 10.3390/plants13172452, PMID: 39273936 PMC11397575

[B43] Kalcsits L. A. Buschhaus H. A. Guy R. D. (2014). Nitrogen isotope discrimination as an integrated measure of nitrogen fluxes, assimilation and allocation in plants. Physiol. Plant 151, 293–304. doi: 10.1111/ppl.12167, PMID: 24512444

[B44] Khan I. Awan S. A. Rizwan M. Brestic M. Xie W. (2023). Silicon: an essential element for plant nutrition and phytohormones signaling mechanism under stressful conditions. Plant Growth Regul. 100, 301–319. doi: 10.1007/s10725-022-00872-3

[B45] Kheyri N. Ajam-Norouzi H. Mobasser H. R. Torabi B. (2019). Effects of silicon and zinc nanoparticles on growth, yield, and biochemical characteristics of rice. Agron. J. 111, 3084–3090. doi: 10.2134/agronj2019.04.0304

[B46] Kim Y. H. Khan A. L. Waqas M. Lee I. J. (2017). Silicon regulates antioxidant activities of crop plants under abiotic-induced oxidative stress: a review. Front. Plant Sci. 8. doi: 10.3389/fpls.2017.00510, PMID: 28428797 PMC5382202

[B47] Kumar V. Kumar S. Dwivedi S. Agnihotri R. Sharma P. Mishra S. . (2025). Combined supplementation of selenium and silica boosts growth and yield of rice (*Oryza sativa* L.) by stimulating photosynthetic efficiency and nutrient uptake. Physiol. Mol. Biol. Plants, 1–21. doi: 10.1007/s12298-025-01592-4, PMID: 41164110 PMC12559515

[B48] Lavinsky A. O. Detmann K. C. Reis J. V. Ávila R. T. Sanglard M. L. Pereira L. F. . (2016). Silicon improves rice grain yield and photosynthesis specifically when supplied during the reproductive growth stage. J. Plant Physiol. 206, 125–132. doi: 10.1016/j.jplph.2016.09.010, PMID: 27744227

[B49] Lee S. Masclaux-Daubresse C. (2021). Current understanding of leaf senescence in rice. Int. J. Mol. Sci. 22, 4515. doi: 10.3390/ijms22094515, PMID: 33925978 PMC8123611

[B50] López J. Way D. A. Sadok W. (2021). Systemic effects of rising atmospheric vapor pressure deficit on plant physiology and productivity. Global Change Biol. 27, 1704–1720. doi: 10.1111/gcb.15548, PMID: 33683792 PMC8251766

[B51] Lu M. (2024). Impact of climate change on rice and adaptation strategies: A review. Adv. Res. Res. 4, 252–262. doi: 10.50908/arr.4.2_252

[B52] Ma J. F. (2003). Functions of silicon in higher plants. Prog. Mol. Subcell. Biol. 33, 127–147. 14518371 10.1007/978-3-642-55486-5_5

[B53] Mathur P. Roy S. (2020). Nanosilica facilitates silica uptake, growth and stress tolerance in plants. Plant Physiol. Biochem. 157, 114–127. doi: 10.1016/j.plaphy.2020.10.011, PMID: 33099119

[B54] Mohamed A. A. E. Zayed B. A. Ghareib H. S. Essa E. A. (2015). The role of silica application in raising rice salinity tolerance and productivity. J. Agric. Res. Kafr El-Sheikh Univ. 41, 232–245.

[B55] Mohapatra P. K. Sahu B. B. (2021). “ Importance of rice as human food,” in Panicle architecture of rice and its relationship with grain filling ( Springer International Publishing, Cham), 1–25.

[B56] Mostofa M. G. Rahman M. M. Ansary M. M. U. Keya S. S. Abdelrahman M. Miah M. G. . (2021). Silicon in mitigation of abiotic stress-induced oxidative damage in plants. Crit. Rev. Biotech. 41, 918–934. doi: 10.1080/07388551.2021.1892582, PMID: 33784900

[B57] Naing A. H. Kim C. K. (2021). Abiotic stress-induced anthocyanins in plants: Their role in tolerance to abiotic stresses. Physiol. Plant 172, 1711–1723. doi: 10.1111/ppl.13373, PMID: 33605458

[B58] Parimala M. Singh J. (2022). Soil and foliar application of silicon on quality parameters and yield of horticultural crops. Pharma Inn. J. 11, 427–433.

[B59] Pavlović D. Nikolić B. Đurović S. Waisi H. Anđelković A. Marisavljević D. (2014). Chlorophyll as a measure of plant health: Agroecological aspects. Pesticidi i fitomedicina 29, 21–34. doi: 10.2298/PIF1401021P

[B60] Ramírez-Olvera S. M. Trejo-Téllez L. I. Gómez-Merino F. C. Ruíz-Posadas L. del Alcántar-González M. E. G. Saucedo-Veloz C. (2021). Silicon stimulates plant growth and metabolism in rice plants under conventional and osmotic stress conditions. Plants 10, 777. doi: 10.3390/plants10040777, PMID: 33920948 PMC8071275

[B61] Rezzouk F. Z. Gracia-Romero A. Kefauver S. C. Gutierrez N. A. Aranjuelo I. Serret M. D. . (2020a). Remote sensing techniques and stable isotopes as phenotyping tools to assess wheat yield performance: effects of growing temperature and vernalization. Plant Sci. 295, 1–16. doi: 10.1016/j.plantsci.2019.110281, PMID: 32534622

[B62] Rezzouk F. Z. Gracia-Romero A. Kefauver S. C. Nieto-Taladriz M. T. Serret M. D. Araus J. L. (2022). Durum wheat ideotypes in Mediterranean environments differing in water and temperature conditions. Agric. Wat. Manage. 259, 107257. doi: 10.1016/j.agwat.2021.107257 PMC8718713 35005145

[B63] Rezzouk F. Z. Shahid M. A. Elouafi I. A. Zhou B. Araus J. L. Serret M. D. (2020b). Agronomic performance of irrigated quinoa in desert areas: Comparing different approaches for early assessment of salinity stress. Agric. Wat. Manage. 240, 106205. doi: 10.1016/j.agwat.2020.106205

[B64] Rohman A. Helmiyati S. Hapsari M. Larasati Setyaningrum D. (2014). Rice in health and nutrition. Int. Food Res. J. 21, 13–24.

[B65] Rouphael Y. Colla G. (2020). Biostimulants in agriculture. Front. Plant Sci. 11. doi: 10.3389/fpls.2020.00040, PMID: 32117379 PMC7010726

[B66] Samal P. Babu S. C. Mondal B. Mishra S. N. (2022). The global rice agriculture towards 2050: An inter-continental perspective. Outlook Agric. 51, 164–172. doi: 10.1177/00307270221088338

[B67] Shahrajabian M. H. Chaski C. Polyzos N. Petropoulos S. A. (2021). Biostimulants application: A low input cropping management tool for sustainable farming of vegetables. Biomolecules 11, 698. doi: 10.3390/biom11050698, PMID: 34067181 PMC8150747

[B68] Shrestha J. Kandel M. Subedi S. Shah K. K. (2020). Role of nutrients in rice (*Oryza sativa* L.): A review. Agrica 9, 53–62. doi: 10.5958/2394-448X.2020.00008.5

[B69] Souri Z. Khanna K. Karimi N. Ahmad P. (2021). Silicon and plants: current knowledge and future prospects. J. Plant Growth Regul. 40, 906–925. doi: 10.1007/s00344-020-10172-7

[B70] Ulloa M. Nunes-Nesi A. da Fonseca-Pereira P. Poblete-Grant P. Reyes-Díaz M. Cartes P. (2021). The effect of silicon supply on photosynthesis and carbohydrate metabolism in two wheat (*Triticum aestivum* L.) cultivars contrasting in response to phosphorus nutrition. Plant Physiol. Biochem. 169, 236–248. doi: 10.1016/j.plaphy.2021.11.022, PMID: 34808466

[B71] Vieira J. F. Nadal M. Tunes L. V. Duarte G. Gewehr E. de Sousa Costa M. (2020). Foliar and time of application of silicon and the effect on rice yield components, productivity and seed quality. Afr. J. Agric. Res. 16, 1721–1725. doi: 10.5897/AJAR2020.14964

[B72] Xu R. Huang J. Guo H. Wang C. Zhan H. (2023). Functions of silicon and phytolith in higher plants. Plant Signal. Behav. 18, 2198848. doi: 10.1080/15592324.2023.2198848, PMID: 37031433 PMC10085572

[B73] Yan W. Li J. Lin X. Wang L. Yang X. Xia X. . (2022). Changes in plant anthocyanin levels in response to abiotic stresses: A meta-analysis. Plant Biotech. Rep. 16, 497–508. doi: 10.1007/s11816-022-00777-7

[B74] Ye D. Wu L. Li X. Atoba T. O. Wu W. Weng H. (2023). A synthetic review of various dimensions of non-destructive plant stress phenotyping. Plants 12, 1698. doi: 10.3390/plants12081698, PMID: 37111921 PMC10146287

[B75] Zellner W. Datnoff L. (2020). “ Silicon as a biostimulant in agriculture,” in Biostimulants for Sustainable Crop Production. Eds. Rouphael Y. du Jardin P. Brown P. De Pascale S. Colla G. ( Burleigh Dodds Science Publishing Limited, Cambridge, UK), 149–195.

[B76] Zhao W. Chou J. Li J. Xu Y. Li Y. Hao Y. (2022). Impacts of extreme climate events on future rice yields in global major rice-producing regions. Int. J. Environ. Res. Public Health 19, 4437. doi: 10.3390/ijerph19084437, PMID: 35457305 PMC9031651

