# Supplementary material for: Interactive effects of silicon formulations, concentrations, and foliar application timing on rice physiology and yield
Source: Front Plant Sci. 2026 Jan 21;16:1723079. doi: 10.3389/fpls.2025.1723079 (PMC12871390; doi:10.3389/fpls.2025.1723079)
Supplement: Supplementary file 1 [file DataSheet1.pdf]

Supplementary Table 1. Microclimatic conditions inside the greenhouse during the experimental period.

	**Temperature (ºC)**	**Relative humidity (%)**
**Average**	24.43	60.57
**Maximum**	13.51	21.50
**Minimum**	44.02	94.49

Supplementary Table 2. Determination of the maximum daily intake (MDI) per person per day and assessment of element acceptability in relation to the maximum thresholds established by EFSA (2009). The MDI was calculated as the product of the average rice consumption (ARC) per person per day and the maximum concentration recorded (MCR) for each element in the different treatments tested in the experiment.

Element	Average rice consumption (ARC) per person and day	Maximum concentration recorded (MCR) in the experiment	Maximum daily intake (MDI) per person and day	Maximum thresholds per element (EFSA 2009)	Acceptable levels of the element
**P**	100 mg Dry weight	0.156 $g\ 100g^{-1}$ DW	156 mg	700 mg	Yes
**S**	100 mg Dry weight	0.148 $g\ 100g^{-1}$ DW	148 mg	Not established	-
**K**	100 mg Dry weight	0.650 $g\ 100g^{-1}$ DW	650 mg	4700 mg	Yes
**Ca**	100 mg Dry weight	0.091 $g\ 100g^{-1}$ DW	91 mg	1000 mg	Yes
**Mg**	100 mg Dry weight	0.160 $g\ 100g^{-1}$ DW	160 mg	370 mg	Yes
**Na**	100 mg Dry weight	0.650 $g\ 100g^{-1}$ DW	650 mg	2300 mg	Yes
**B**	100 mg Dry weight	9.56 $mg\ Kg^{-1}$ DW	0.956 mg	Not established	-
**Cu**	100 mg Dry weight	12.80 $mg\ Kg^{-1}$ DW	1.280 mg	0.9 mg	No
**Fe**	100 mg Dry weight	30.49 $mg\ Kg^{-1}$ DW	3.049 mg	13.0 mg	Yes
**Zn**	100 mg Dry weight	62.73 $mg\ Kg^{-1}$ DW	6.273 mg	10.0 mg	Yes
**Mn**	100 mg Dry weight	13.16 $mg\ Kg^{-1}$ DW	1.316 mg	2.1 mg	Yes
**Mo**	100 mg Dry weight	2.42 $mg\ Kg^{-1}$ DW	0.242 mg	Not established	-
**Co**	100 mg Dry weight	0.45 $mg\ Kg^{-1}$ DW	0.045 mg	2.5 mg	Yes
**Ni**	100 mg Dry weight	0.80 $mg\ Kg^{-1}$ DW	0.080 mg	Not established	-
**Pb**	100 mg Dry weight	0.36 $mg\ Kg^{-1}$ DW	0.036 mg	Not established	-
**Cd**	100 mg Dry weight	0.071 $mg\ Kg^{-1}$ DW	0.007 mg	Not established	-
**Cr**	100 mg Dry weight	0.58 $mg\ Kg^{-1}$ DW	0.058 mg	0.125 mg	Yes
**Rb**	100 mg Dry weight	2.93 $mg\ Kg^{-1}$ DW	0.293 mg	Not established	-
**Si**	100 mg Dry weight	0.72 $mg\ Kg^{-1}$ DW	0.072 mg	Not established	-
**Li**	100 mg Dry weight	0.58 $mg\ Kg^{-1}$ DW	0.058 mg	Not established	-
**Sr**	100 mg Dry weight	2.61 $mg\ Kg^{-1}$ DW	0.261 mg	Not established	-
**Ti**	100 mg Dry weight	1.07 $mg\ Kg^{-1}$ DW	0.107 mg	Not established	-
**Tl**	100 mg Dry weight	0.82 $mg\ Kg^{-1}$ DW	0.082 mg	Not established	-
**Bi**	100 mg Dry weight	1.43 $mg\ Kg^{-1}$ DW	0.143 mg	Not established	-
